# Synchronous diffuse ganglioneuromatosis and multiple schwannomas of the colon: A case report and literature review

**DOI:** 10.3892/etm.2015.2212

**Published:** 2015-01-23

**Authors:** CHANGLI LU, YAN QIU, XUFENG LU, GANDI LI, HONG BU

**Affiliations:** 1 Department of Pathology, West China Hospital, Sichuan University, Chengdu, Sichuan 610041, P.R. China; 2 Regenerative Medicine Research Center, West China Hospital, Sichuan University, Chengdu, Sichuan 610041, P.R. China

**Keywords:** ganglioneuromas, diffuse ganglioneuromatosis, schwannoma, neurofibromatosis

## Abstract

Diffuse ganglioneuromatosis (DG) of the gastrointestinal tract is a rare condition that is closely associated with neurofibromatosis type 1 and multiple endocrine neoplasia type 2B. The occurrence of DG with multiple schwannomas, which, of the GI tract, usually affect the stomach, is considerably more rare. The present study describes the case of a 54-year-old male with indolent DG, principally involving the small intestine and colon, associated with multiple schwannomas in the subserosa. The patient was treated with surgery. A brief overview of intestinal ganglioneuromatous lesions and the associated conditions is additionally presented.

## Introduction

Ganglioneuromas (GNs) of the gastrointestinal (GI) tract are rare tumors characterized by hyperplasia of ganglion cells, nerve fibers and supporting cells. Shekitka *et al* (1) divided GNs into three groups: Polypoid GN, ganglioneuromatous polyposis (GP) and diffuse ganglioneuromatosis (DG). Polypoid GN is the most common type, a benign solitary polyp involving the mucosa and submucosa that resembles an adenoma or a juvenile polyp. GP is usually distinguished by numerous discrete sessile or pedunculated mucosal and/or submucosal lesions mimicking familial adenomatous polyposis, which may be associated with multiple cutaneous lipomas and a family history of multiple intestinal polyps (2). DG is characterized by a transmural proliferation of the neural plexus in the bowel wall and is closely associated with neurofibromatosis type 1 (NF 1) (3) and multiple endocrine neoplasia type 2B (MEN 2B) (4). Schwannomas of the GI tract have been reported relatively rarely and have occurred predominantly in the stomach, accounting for 3.3–12.8% of all GI mesenchymal tumors (5,6). DG with multiple schwannomas is a rare condition. It is yet to be elucidated whether the occurrence of DG with multiple schwannomas is incidental or whether the two lesions are connected through a causal association. The present case report describes a male with DG of the GI and schwannomas. In combination with the relevant literature, the diagnosis and treatment of the patient are discussed in the present study.

## Case report

### Patient history

In October 2012, a 54 year-old Chinese male was admitted to West China Hospital (Chengdu, China) with a one-month history of intermittent bloody stools and abdominal pain, without diarrhea or vomiting. The colonoscopy revealed >50 sessile, bead-like polyps ranging grossly in size from 0.1 to 8 cm throughout the entire colon. The patient also underwent an esophagogastroduodenoscopy to exclude other similar lesions in the GI. Endoscopic examination and biopsy specimens from the gastric cardia revealed no specific histopathology. No pigmented skin lesions were identified on physical examination. Tumor marker studies revealed that calcitonin, α-fetoprotein, carcinoembryonic antigen and cancer antigen 19-9 levels were normal.

The patient gave a medical history of two previous laparotomies in a local hospital. The first time was 43 years previously when the patient was 11 years old. At this time, the patient was admitted to a local hospital with colicky abdominal pain, vomiting and the passage of bloody stools. From the laparotomy, a small intestinal intussusception was identified and reduced. Resection of a segment of the small intestine and a primary anastomosis were carried out. A polyp was found in the small intestine but the patient could not remember any details of the pathological diagnosis.

The second laparotomy was eight years previously at the age of 46. The patient was admitted to a local hospital with abdominal pain. The colonoscopy showed multiple polyps in the small intestinal, which were removed by surgery. Since the pathological change was uncommon, the slices of specimen were transferred to West China Hospital for consultation.

There was no history of polyposis or colonic disease among the parents, siblings or children of the proband. The patient and his family had no known history of familial adenomatous polyposis, NF 1, MEN 2B or Cowden syndrome (CS).

### Diagnosis

**Figure 1:**
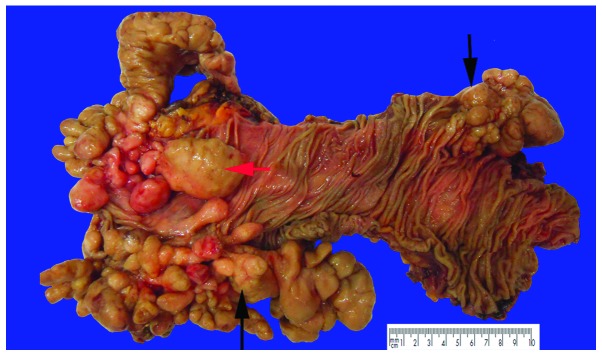
Partial colectomy specimen. Black arrows show small sessile polyps, linked together to produce stricture-like thickenings of segments of the bowel. The red arrow shows larger pedunculated polyps, ranging from 1 to 5.2 cm in diameter, forming large irregular, nodular lesions. The overlying mucosa between the lesions was intact.

**Figure 2:**
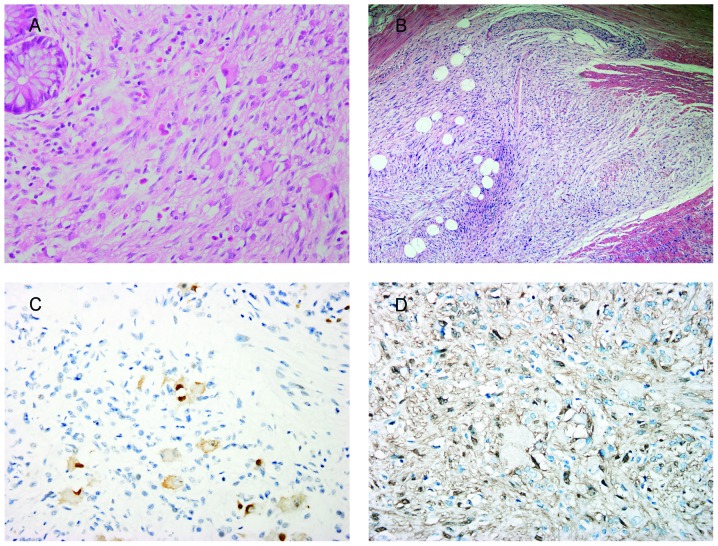
Histology of diffuse ganglioneuromatosis. (A) Ganglion and nerve sheath cell proliferation was predominantly found in the lamina propria and submucosa (HE staining; magnification, ×400). The ganglion cells were large, with rich eosinophilic cytoplasm, enlarged nuclei and prominent nucleoli. (B) Plexiform or band-like enlargement of the nerve fibers and ganglion cells was observed in the myenteric plexuses (HE staining; magnification, ×100). (C) Ganglion cells were positive for NeuN, while the nerve fibers were negative for NeuN (IHC staining; magnification, ×400). (D) Nerve fibers were positive for S100, while the ganglion cells were negative for S100 (IHC staining; magnification, ×400). HE, hematoxylin and eosin; IHC, immunohistochemical; NeuN, neuron-specific nuclear protein.

The specimen that was sent to the hospital consisted of the ascending, transverse and descending colons of the splenic flexure. The specimen measured 30 cm in length. There was a diffuse thickening of the intestinal wall and no evidence of perforation. Numerous (50 to 80) sessile or pedunculated polyps ranging in size from 0.1 to 8 cm in the colon were observed. The sessile polyps were small, linked together and hard to count, and produced stricture-like thickenings of segments of the bowel. By contrast, the pedunculated polyps were large, with diameters ranging from 1 to 5.2 cm, and formed large, irregular, nodular lesions. The overlying mucosa between the lesions was intact (Fig. 1). Two histological growth patterns were identified: i) The proliferation of ganglion and nerve sheath cells was mainly found in the lamina propria and submucosa (Fig. 2A), and constituted the predominant lesions of the colon; ii) a plexiform or band-like enlargement of the nerve fibers and ganglion cells was observed in the myenteric plexuses (Fig. 2B). The ganglion cells were large, with rich eosinophilic cytoplasm, enlarged nuclei and prominent nucleoli; however, no significant nuclear pleomorphism or mitotic activity was noted. In addition, large numbers of eosinophils and sparse histiocytes, lymphocytes and neutrophils were observed. The lesion was transmural, without the involvement of the subserosa or the serosal fat. Immunohistochemical (IHC) stains revealed that the ganglion cells were positive for neuron-specific nuclear protein (NeuN) (Fig. 2C), neurofilament (NF) and neuron-specific enolase, while the nerve fibers were negative for NeuN and cluster of differentiation (CD) 117, but positive for NF (Fig. 2D) and S100. The positive rate of Ki67 in the ganglion cells and nerve fibers was <2%. A review of the hematoxylin and eosin and IHC slices from eight years previously, which showed similar findings, also confirmed the pathological diagnosis of DG.

**Figure 3:**
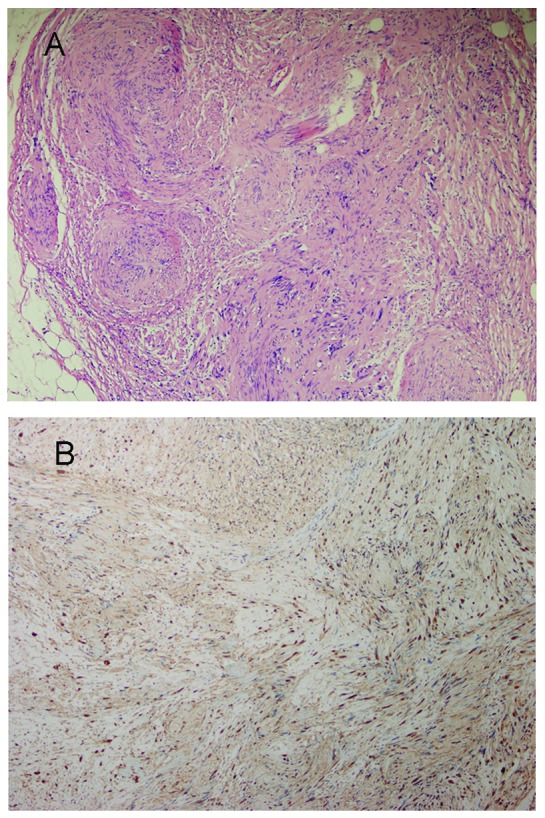
(A) Nodule of schwannoma without a capsule in the subserosa, showing features typical of Antoni A (cells forming a typical palisade arrangement in a well-organized pattern) and Antoni B (loose cells without palisade architecture) regions (hematoxylin and eosin staining; magnification, ×100). (B) Spindle cells were positive for S100 protein (immunohistochemical staining; magnification, ×200).

In addition to the DG, >30 nodules of schwannomas without capsules, ranging in size from 0.1 to 1.5 cm, were found in the subserosa, some even involving the surrounding adipose tissue. These showed typical features of schwannoma, with Antoni A (cells forming a typical palisade arrangement in a well-organized pattern) and Antoni B (loose cells without palisade architecture) regions in variable proportions (Fig. 3A) and cells with strong positivity for S100 protein (Fig. 3B) but negative results for CD117, discovered on gastrointestinal stromal tumors protein 1 (DOG1), desmin, smooth muscle actin (SMA), CD34, CgA and pancytokeratin, as determined using IHC assay.

### Treatment and follow-up

The patient underwent surgery and made an uneventful recovery. The patient also received Traditional Chinese Medicine to coordinate the intestines and stomach, but was not administered any other treatments. A follow-up observation with repeat endoscopic evaluation was performed once every six months; two years later the patient showed no evidence of recurrence and was in good health.

## Discussion

**Figure 4:**
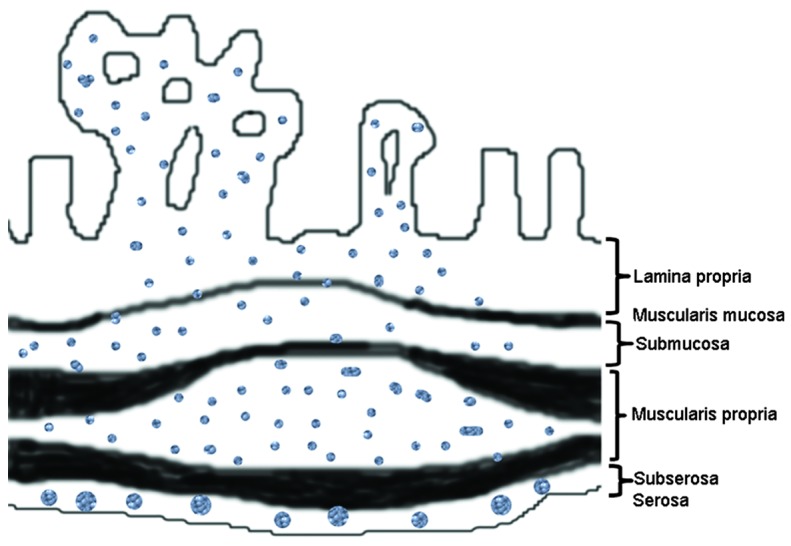
Schematic overview of DG and schwannomas. Ganglioneuromatous polyposis is characterized by aggregates of ganglion cells and nerve fibers within the colonic mucosa, while DG can be mucosal or transmural with a diffuse, band-like enlargement of the nerve fibers and ganglion cells of the submucosal and myenteric plexuses. The present case combined these two patterns of histological change together with involvement from the lamina propria to the muscularis propria. At the same time, >20 nodules of schwannomas without capsules, ranging in diameter from 0.1 to 1.5 cm, were found in the subserosa of the colon.

GP is characterized by aggregates of ganglion cells and nerve fibers within the colonic mucosa, while DG can be mucosal or transmural with a diffuse, band-like enlargement of nerve fibers and ganglion cells of the submucosal and myenteric plexuses and more pronounced changes in the latter (1,7). Notably, the present case appeared to combine the two patterns of histological change together with involvement from the lamina propria to the muscularis propria (Fig. 4). The condition was classified as DG due to its transmural growth pattern and giant polyps. The intramural growth pattern and florid hyperplasia of the submucosal or myenteric plexus are distinct to DG and often occur with NF 1 and the first manifestations of MEN 2B.

NF 1, also named Von Recklinghausen’s disease, is an autosomal dominant disease characterized by mucocutaneous neurofibromas and café-au-lait spots and involves numerous organs, including the GI (8). NF 1 is caused by the mutation of a gene on chromosome 17 that is responsible for the control of cell division. The patient in the present case showed no skin pigment deposition or skin neurofibromas, and no osseous lesion or optic glioma by computed tomography. MEN 2B (medullary thyroid carcinoma, pheochromocytoma, oral mucosal neuromas and skeletal deformities) typically manifests before a child reaches 10 years of age. Variations in the RET proto-oncogene cause MEN 2B, and DNA analysis for M918T mutation is the preferred method of establishing the diagnosis (9,10). The patient in the present case had none of the manifestations associated with NF 1 or MEN 2B.

The patient had a long disease history of 43 years. Although there was insufficient pathological evidence to support a diagnosis of DG when the patient was 11 years old, the possibility of DG could not be excluded. The patient underwent three surgical procedures due to polyps; the main symptoms were abdominal pain and bloody stools. The recurrence of the DG may have been associated with the extensive involvement of the lesions and incomplete excision. The lesions were so extensive that it was difficult to achieve a complete excision, even in the third surgery. The growth index, Ki67, of the ganglion cells and nerve fibers was <2%, and no mitotic figures or nuclear pleomorphism were observed, which suggested that DG exhibits an indolent or benign biological behavior. Malignant transformation of DG itself has not been reported, to the best of our knowledge; however, there are a number of case reports of DG coincident with adenocarcinoma (11–14), carcinoid tumor (15) or malignant peripheral nerve sheath tumor (1,16). Kanter *et al* (17) suggested that DG should be considered as a premalignant condition, but this association is controversial (13,18). Genetic testing has revealed PTEN mutations in a handful of DGs with CS. Heald *et al* (19) reported that nine (13%) out of a total of 65 patients undergoing colonoscopy who were PTEN mutation carriers were diagnosed with colorectal cancer, all before the age of 50 years; however, the pathogenesis of these ganglioneuromatous lesions is yet to be elucidated. It is not known whether or not such an association with malignant tumors is a simple incidental coexistence or whether the two lesions are connected by a causal association.

Schwannomas in the GI tract are rare, while occurrence in the colon is extremely rare (20). Schwannomas are derived from the Schwann cells that form the neuronal sheath (21), while DG is believed to represent an unusual hyperplasia of the nerve plexuses, including a mixed hyperplasia of three types of cells (neuronal cells, nerve fibers and supporting cells). The presence of ganglion cells in DG makes the condition notably different from schwannoma. In general, schwannomas behave in a benign manner; however, they have the tendency to recur locally and become malignant if left untreated (5,22). Occasionally, benign schwannomas may invade several layers of the bowel wall and even involve the surrounding adipose tissue (22). The immunophenotype of schwannomas differs from that of other intestinal mesenchymal neoplasms, such as smooth muscle tumors, GI stromal tumors and neurofibromas. This difference in immunophenotype is represented by the spindle cells of schwannomas showing strong diffuse positivity for S100 and an absence of staining for CD117, DOG1, CD34, desmin, SMA and actin. With regard to neurofibromas in the GI, tumors consist of a mixture of spindle cells with wavy nuclei and strands of collagen, as well as Schwann cells and perineural fibroblasts. Tumor cells are also weakly positive for S100. Wavy nuclei and strands of collagen can facilitate differentiation (23).

Treatment of GNs depends on the number, size, anatomical location and clinical history (24). Polypectomy with hot biopsy forceps is curative for polypoid GN; therefore, radical surgery is the accepted standard treatment of GP and DG. Radiotherapy or chemotherapy is not recommended. Due to the association that exists between DG and other systemic diseases, it has been suggested that patients with DG should undergo careful screening for malignancies in the colon and elsewhere, as well for the two associated syndromes (25); however, certain authors have stated that this screening is unnecessary due to the benign nature, slow-growing and indolent behavior of DGs (26). Furthermore, screening could lead to over-treatment, increased financial costs and health risks due to increased endoscopic surveillance.

In conclusion, the present study describes the case of a male with recurrent DG of the GI who developed multiple schwannomas in the subserosa of the colon. To the best of our knowledge, this case report of the synchronous occurrence of DG and schwannomas in the colon is extremely rare. The nature and significance of this association are unclear. The two diseases are slow growing and well-differentiated neurogenic neoplasias. In this particular case, it was not known whether there was an association between the DG and schwannomas or whether their coexistence was coincidental. Further studies are required to clarify the molecular alterations in such cases and reveal the etiology of this association.
